# The effect of high medical expenses on household income in South Korea: a longitudinal study using propensity score matching

**DOI:** 10.1186/s12913-015-1035-5

**Published:** 2015-09-10

**Authors:** Jae Woo Choi, Eun Cheol Park, Ki Bong Yoo, Sang Gyu Lee, Sung In Jang, Tae Hyun Kim

**Affiliations:** Department of Public Health, Yonsei University College of Medicine, Seoul, Republic of Korea; Institute of Health Services Research, Yonsei University College of Medicine, Seoul, Republic of Korea; Department of Preventive Medicine, Yonsei University College of Medicine, Seoul, Republic of Korea; Department of Hospital Administration, Graduate School of Public Health, Yonsei University, Seoul, Republic of Korea

## Abstract

**Background:**

Almost 97 % of the Korean population is covered by National Health Insurance and are entitled to receive the same level of insurance benefits, regardless of how much each enrollee contributes to the system. However, the percentage of out-of-pocket payments is still high. This study examines whether the incurrence of high medical expenses affects household income.

**Methods:**

We use the Korea Welfare Panel and select 4,962 households to measure repeatedly over 5 years. Using propensity score matching, we set households with medical expenses of three times the annual average as “occurrence households” while “non-occurrence households” are those below the cut-off but with all other factors, such as income, held constant. We analyze whether the income of occurrence households differs significantly from the comparison group using a linear mixed effect model.

**Results:**

After the occurrence of high medical expenditure, occurrence households (*n* = 825) had US$ 1,737 less income than non-occurrence households. In addition, the income of households (*n* = 200) that incurred high medical costs repeatedly for 2 years was US$ 3,598 lower than the non-occurrence group.

**Conclusions:**

Although it is important for the government to focus on medical assistance for households that have medical expense burdens, it needs to consider providing income indemnity insurance to protect them.

## Background

The number of households in Korea that have declared bankruptcy has been on the increase recently due to excessive medical expenditure. Major causes of this situation include low benefits and a high co-payment rate [1]. The Korean government invested tremendous resources between 2005 and 2008 in order to increase the health insurance payment rate to 70 % of costs as part of an initial Health Insurance Benefit Strengthening Plan. Subsequently, between 2009 and 2013, the second Health Insurance Benefit Strengthening Plan was established [2]. However, the level of coverage is only 55.3 % in Korea compared to an average of 72.2 % in Organisation for Economic Co-operation and Development (OECD) countries in 2011. Korea’s public expenditure rate has fallen every year since 2009—from 56.7 % in 2009, to 56.5 % in 2010, and 55.3 % in 2011—and we postulate that this is responsible for the falling health insurance benefit rates of 65 % in 2009, 63.6 % in 2010, and 63 % in 2011. When the benefit rate decreases, the burden on households inevitably rises, causing household health expenditure to increase. Compared to 34.2 % in 2010, the co-payment rate for households increased by 1 percentage point in 2011 to 35.2 % [3].

Most research on the effects of changes in Korean health insurance has proved that insurance has an insignificant effect on the co-payment burden [4]. The insufficient level of financial security means that families and individuals have a significant health expense burden, leading to substantial financial difficulties [5, 6]. In fact, the medical costs of households comprised 35.2 % of all household expenditure in Korea in 2011, which is much higher than the OECD average of 19.6 % in 2011. This poses severe problems for households at the lowest income levels [7].

High medical expenditure result in not only reduced ability to pay for basic needs, such as food and medicine, but also reduced ability to spend on education [8]. Furthermore, many households fall into poverty owing to the high co-payment level [9, 10]. In a polarizing social structure, the loss of one’s health signifies much more than a simple temporal loss to those whose labor power relies heavily on good health. Illness represents loss of working ability and, at least in a capitalist society, can mean job loss and lead directly to social poverty [11, 12]. The higher the risk of illness is for lower income tiers, the higher is the probability that the state is not functioning properly. The government has the responsibility to protect the weak according to the principals of social justice [13] and on this point, the security of the social safety net is an important factor to consider. To strengthen the national social security net effectively with limited resources, the government can support households going through illness-related risk above a certain level by measuring the level of catastrophic medical expenditure rate of households.

There are two methods to determine the standard of catastrophic medical expenditure incurred by households. The first is the relative concept in which medical spending exceeds a certain percentage of a household’s total income or spending [14–19]. The second is the absolute concept in which excessive medical expenditure is defined as medical spending that exceeds a set amount [20].

Although there have been many studies related to households’ catastrophic medical expenditure that have employed the relative concept of the catastrophic medical expenditure index [21–23], research utilizing the absolute concept has been minimal [24–26]. Using an absolute measure of catastrophic medical expenses has a few limitations when defining high medical costs. Although the payment of US$ 1,000 would be a burden for a low-income individual, it might not be as big a problem for a high-income individual. However, many patients and their families still end up paying substantial amounts of medical expenses (e.g., US$ 1,000 dollars or more) even though almost of all of them are covered by Korea’s National Health Insurance (NHI) scheme. Enrollees with lower income pay the same amount of out-of-pocket (OOP) payments although they contribute lower insurance premiums. Based on these conditions in South Korea, we consider using an absolute measure of high medical expenses. Thus, we utilized a mixed method, which calculates absolute price with reference to average medical costs of the population, which is a relative measure. Average medical costs have been used mainly as an index of representative value and differ from previous studies, which have used the absolute measure only.

According to Korea’s NHI, 15 % of patients accounted for more than 50 % of total medical expenses, which illustrates the fact that a minority of patients with high medical expenditure are the biggest consumers of insurance benefits. In addition, the Health Insurance Statistics Analysis Source book (2010) states that the number of high-cost patients whose medical expenditure exceed US$ 5,000 increased from 209,305 in 2000 to 1,017,222 in 2009, which is almost a five-fold increase and shows that the risk of high medical costs is increasing [27].

We can assume that high medical expenditure lead to a vicious cycle of poverty [28]. However, there have been no clear findings on the extent to which high medical expenditure affects household income. Therefore, this research aims to examine whether high medical costs affects household income.

## Methods

### Research model

The KWP surveys working income, business and sideline business income, property income, private transfer income, public transfer income, and non-recurring income (other income) every year. The present study used disposable income (working income + business and sideline business income + property income + private transfer income + public transfer income − taxation and social security burden charge) as the dependent variable. Then, we used the calculated variable of the monthly household income average multiplied by 12 as the altered annual household income. We used medical expenses that are purely OOP payments. OOP costs were defined as the average annual direct payment for hospitalization, outpatient visits, dental treatment, surgery, prescription drugs, nursing care, and health examination. This study set independent variables as the main factors, such as medical needs, payment ability, and other demographic characteristic variables. Medical needs comprised the number of elderly above the age of 65 years and the number of family members with chronic disease. Individuals with chronic illness were characterized in the survey as those who suffered from one or more chronic diseases for more than 6 months. Payment ability comprised the number of employees, regardless of whether the householder and other household members were employed. Householder’s characteristics (sex, age, education level, marriage status) were included in the other demographic characteristic variables. The householder represents his or her household by bearing responsibility for the household’s livelihood and influencing the economic decisions of the household [29]. Therefore, we included demographic characteristics of the householder to reflect the household features.

### Data source

This study used Korea Welfare Panel 3^rd–^7^th^ (2007^−^2011 subjects) data co-produced by the Korea Institute for Health and Social Affairs and the Seoul National University Social Welfare Research Center. This survey was conducted with the goal of understanding the population group’s living conditions and welfare demands, and evaluating the effectiveness of policies to utilize in the formation of policies. The biggest strength of the Korea Welfare Panel data is that it has both time-series and cross-sectional features.

Korea Welfare Panel data was collected using an interview research method in which the interviewer questioned and recorded the answers of the interviewees. A stratified cluster systematic method was used to select the sample of households for the research, and this included the data of the 3^rd^ (6,128 households), 4^th^ (5,935 households), 5^th^ (5,675 households), 6^th^ (5,335 households), and 7^th^ (5,735 households) research periods. This study excluded households that were omitted or added during the 3^rd^ to 7^th^ research periods, and thus, used 4,962 households. The current study was approved by the Institutional Review Board of Yonsei University Graduate School of Public Health.

### Analysis methods

The average annual medical expenditure of households that were surveyed in the 3^rd^ Korea Welfare Panel dataset were about US$ 1,000. This study selected households spending more than three times the average annual household medical expenditure; these households were defined as “high medical expenditure occurrence households.” We defined “non-occurrence households” as having similar characteristics, including the same income levels, but without high medical expenditure.

We used propensity score matching (PSM) to solve selection bias between groups in this study. PSM reduces the heterogeneous nature between experimental and control groups and assumes similar conditions for randomization of the experimental design. The methods available for matching are nearest neighbor matching, radius matching, and kernel matching. We chose nearest neighbor matching for our analysis [30]. Healthcare expenditure include hospital fees, outpatient medical examination fees, dental care fees, surgery fees, medicine costs, health checkup fees, health supplementary food, and purchases of health and medical care goods (e.g., glasses and contact lenses). We verified whether income differences would occur depending on predictable factors for each group by conducting a chi-square test. The factors of medical needs, payment ability, and other demographic characteristic variables were controlled to analyze whether there was a significantly statistical income difference after the occurrence of high medical expenditure using the linear mixed model. The same model was used to analyze the effects of repeated occurrences of high medical expenditure on the income of households between 2009 and 2011, as there were repeated high medical expenditure during 2007 and 2008. Household income was adjusted by taking the square root of the number of household members. Since prices change annually, the consumer price index was reflected in both income and medical expenditure from 2007 to 2010. As the currency in our data is the Korean won, we converted the US dollar to the Korea won using an exchange rate of US$ 1 = KRW 1,011).

## Results

### General household characteristics

Table 1 shows the results of the Korea Welfare Panel surveys for the 4,962 households that responded to all 3^rd^ to 7^th^ research periods. The data show the general characteristics of 275 households with high medical expenditure (more than three times average annual medical expenditure) and 825 households with similar features but without high medical expenditure.

**Table 1:** General characteristics

								Unit: N, %
Variables	Before propensity score matching					After propensity score matching^a^		
	High medical expense occurrence household		High medical expense non-occurrence household		*P*-value	High medical expense non-occurrence household		*P*-value
Total	275	100.0	6,020	100.0		825	100.0	
Householder level								
Sex					0.8032			0.4369
Man	205	74.5	4,447	73.9		634	76.8	
Woman	70	25.5	1,573	26.1		191	23.2	
Age (unit: years)					<.0001			0.1537
Under 39	29	10.5	1,146	19.0		60	7.3	
40 ~ 64	76	27.6	2,524	41.9		276	33.5	
Over 65	170	61.8	2,350	39.0		480	58.2	
Education level					0.0372			0.3835
Below elementary school	115	41.8	2,103	34.9		297	36	
Middle school	35	12.7	767	12.7		114	13.8	
High school	72	26.2	1,757	29.2		244	29.6	
Above university	53	19.3	1,393	23.1		170	20.6	
Marriage status					0.0162			0.572
Married	196	71.3	3,895	64.7		574	69.6	
Unmarried	11	4	375	6.2		27	3.3	
Divorce, Separated	66	24	1,725	28.7		223	27	
Household level								
Employment of household					<.0001			0.9714
None	104	37.8	1,577	26.2		313	37.9	
>1 person	171	62.2	4,443	73.8		512	62.1	
Senior of household					<.0001			0.1054
None	82	29.8	3,284	54.6		290	35.2	
>1 person	193	70.2	2,736	45.4		535	64.8	
Member with chronic disease					<.0001			0.7451
None	38	13.8	2,260	37.5		110	13.3	
1 person	141	51.3	2,229	37.0		424	51.4	
≥2 people	96	34.9	1,531	25.4		291	35.3	

^a^The results after matching propensity scores for age ∙ sex ∙ education level ∙ marriage status (individual level), employment ∙ senior ∙ member with chronic disease (household level)

In households with high medical expenditure (referred to henceforth as occurrence households), there were 205 male householders (74.5 %) and 70 female householders (25.5 %) while for households without high medical expenditure after matching (referred to as non-occurrence households), there were 634 male householders (76.8 %) and 191 female householders (23.2 %). In terms of householders’ age, occurrence households had the highest percentage of householders (61.8 % or 170 households) aged more than 65 years while non-occurrence households had 58.2 % or 480 householders aged more than 65 years. With regard to education levels, in occurrence households, 115 householders (41.8 %) had the highest portion with no education or primary education while in non-occurrence households, the highest proportion had elementary school education (297 householders or 36 %).

With regard to marriage status, both groups of households mainly included married couples, with 196 married (71.3 %) in occurrence households and 574 married (69.6 %) in non-occurrence households.

Regarding household characteristics, 171 occurrence households (62.2 %) replied that they had more than or equal to one employed members, and 512 non-occurrence households (62.1 %) replied that they had more than or equal to one employed members. In addition, 193 occurrence households (70.2 %) replied that they had more than one elderly member aged more than 65 years and were 535 non-occurrence households (64.8 %) replied that they had more than one elderly member aged more than 65 years. Lastly, 237 occurrence households (86.2 %) replied that they had more than one chronically sick member and 715 non-occurrence households (86.7 %) reported the same. All these results showed no statistically significant difference.

### Changes in average medical expenditure and household income

Table 2 shows the average annual medical expenditure and income of occurrence and non-occurrence households. We set a similar level of income with no statistical difference between the two groups in 2007. The average annual income of occurrence households in 2007 was US$ 19,281 and of non-occurrence households was US$ 18,171, a difference of US$ 1,110, which was not significant. The same trend continued until 2011. By comparison, average annual medical expenditure of occurrence households with high medical expenditure was US$ 9,193 compared to US$ 784 for non-occurrence households, showing a great difference of about US$ 8,409. After 2007, occurrence households trended toward a reduction in this gap— US$ 2,007 in 2008, US$ 747 in 2009, US$ 588 in 2010, and US$ 513 in 2011—but a substantial statistical difference remained.

**Table 2:** Household average income and medical expense

										Unit: USD^a^	
		2007		2008		2009		2010		2011	
		Average	*p*-value	Average	*p*-value	Average	*p*-value	Average	*p*-value	Average	*p*-value
Medical expense	(1)	9,193	0.0100	3,024	<.0001	1,839	<.0001	1,839	0.0001	1,736	0.0007
	(2)	784		1,017		1,092		1,251		1,223	
Income	(1)	19,281	0.3030	17,937	0.4118	17,704	0.1926	19,179	0.7684	18,871	0.8473
	(2)	18,171		18,721		19,048		19,487		18,656	

(1): high medical expense occurrence household

(2): high medical expense non-occurrence household

^a^1 USD = 1,011 KRW

### Factors associated with household income

A mixed effect model was applied to find factors affecting income of households (Table 3). With other independent variables adjusted, the income of high medical expense occurrence households decreased significantly by US$ 1,784 compared to its 1:3 counterpart of 825 non-occurrence households. After the occurrence of high medical expenditure in 2007, occurrence households faced a decrease in their income by US$ 1,737 in 2008, and US$ 2,357 in 2009 compared to non-occurrence households.

**Table 3:** Factors associated with household income

			Unit: USD
Variables	Estimate^§^	Std. Error	*p*-value
Householder level			
Sex			
Man	0		
Woman	−553	86.2	0.4914
Age			
Under 39	0		
40 ~ 64	329	101.2	0.7285
Over 65	−3,887	101.2	0.0037
Education level			
Below elementary school	0		
Middle school	2,081	93.7	0.0175
High school	5,202	85.4	<.0001
above university	11,689	101.7	<.0001
Marriage status			
Married	0		
Unmarried	−7,928	153	<.0001
Divorce, Separated	−1,605	77.7	0.0271
Household level			
Employment of household			
None	0		
>1 person	4,908	43.3	<.0001
Senior of household			
None	0		
>1 person	−2,061	99.2	0.0261
Member with chronic disease			
None	0		
1 person	−225	32.1	0.5691
≥2 people	−1,107	56.8	0.0351
Year (reference: 2007)			
2008	1,408	65.6	0.0216
2009	1,723	101.7	0.0698
2010	197	76.8	0.7836
2011	103	80.4	0.8914
Case (reference: non-occurrence household)			
Occurrence household	−1,784	95.7	0.0458
Interaction term (year x Case)			
2008	−1,737	75.7	0.0142
2009	−2,357	117.3	0.0317
2010	−1,426	86.4	0.0840
2011	−663	92.4	0.4422

§ Adjusted odds ratio from multiple regression analysis with all of the variables in Table 1

The age, education levels, and marriage status of householders, the number of employed household members, elderly household members aged more than 65 years, and members with chronic disease were confirmed as factors of influence on household income.

Households with one or more working members had higher incomes by an average of US$ 4,908 than households without any employed members. Households living with one or more seniors aged more than 65 years had lower incomes by an average of US$ 2,061 than households without any senior citizen. Households living with two people with chronic disease had lower incomes by an average of US$ 1,107 than households without any members with chronic disease.

Considering other demographic characteristic factors, householders aged more than 65 years had on average US$ 3,887 lower income than householders aged 39 years or below. Householders with high school or university levels of education had higher average incomes— US$ 5,202 and US$ 11,689—than householders with no education or only elementary education, respectively. Single or divorced/separated heads of households had income of US$ 7,928, which was US$ 1,605 less than married householders.

### Difference in household income according to occurrence of high medical expenditure

To find income differences that were dependent on high medical expenditure, a linear mixed effect model was used to determine household spending at twice, four times, and five times the average annual medical expenditure and comparative households (Table 4). There were 489 households with double the average medical expenditure and these were set as occurrence households. In addition, non-occurrence households were selected by PSM for household spending that was three times the average medical expenditure. When other independent variables were adjusted, occurrence households had about US$ 1,435 less income compared to non-occurrence households. After the occurrence of high medical expenditure, occurrence households had US$ 1,353 less income than non-occurrence households in 2008. Households (*n* = 174) with four times the average medical expenditure were set as occurrence households. The occurrence households had US$ 2,373 less income than their comparative group. After the occurrence of high medical expenditure in 2007, occurrence household income was US$ 2,233 and US$ 2,862 lower than the non-occurrence group in 2008 and 2009, respectively. Households (*n* = 125) with five times the average medical expenditure were set as occurrence households and their income was US$ 2,553 less than their comparative group. After the occurrence of high medical expenditure in 2007, the income of occurrence households was US$ 2,147, US$ 2,385, and US$ 2,749 lower than the non-occurrence group in 2008, 2009, and 2010, respectively.

**Table 4:** Income difference in occurring high medical expenditure

			Unit: USD
Variables	Estimate^§^	Std. Error	*p*-value
≥ at twice the average medical expense (*n* = 1,956)			
Year (reference: 2007)			
2008	556	44.6	0.1818
2009	47	58.6	0.9315
2010	672	49.8	0.1482
2011	791	55.7	0.1283
Case (reference: non-occurrence household)			
Occurrence household	−1,435	67.6	0.0230
Interaction term (year x Case)			
2008	−1,353	51.5	0.0049
2009	−888	67.5	0.1591
2010	−770	57.3	0.1503
2011	−434	64.0	0.4672
≥ at four times the average medical expense (*n* = 696)			
Year (reference: 2007)			
2008	2,597	103.4	0.0073
2009	2,605	130.7	0.0330
2010	835	107.0	0.4029
2011	1,212	113.6	0.2532
Case (reference: non-occurrence household)			
Occurrence household	−2,373	147.6	0.0453
Interaction term (year x Case)			
2008	−2,233	119.3	0.0453
2009	−2,862	150.6	0.0420
2010	−1,790	123.1	0.1195
2011	−1,342	130.5	0.2710
≥ at five times the average medical expense (*n* = 500)			
Year (reference: 2007)			
2008	2,204	82.9	0.0046
2009	2,340	103.4	0.0157
2010	1,476	107.0	0.1403
2011	1,098	120.8	0.3306
Case (reference: non-occurrence household)			
Occurrence household	−2,553	137.0	0.0464
Interaction term (year x Case)			
2008	−2,147	95.7	0.0167
2009	−2,385	119.0	0.0323
2010	−2,749	123.0	0.0170
2011	−1,840	138.7	0.1556

§ Adjusted odds ratio from multiple regression analysis with all of the variables in Table 1

### Difference in household income according to reoccurrence of high medical expenditure

To establish whether income differences were dependent on the repeated occurrence of high medical expenditure, a linear mixed effect model was used on divided groups of households spending twice or three times the average annual medical expenditure and those households without high medical expenditure (Table 5). Households spending twice the average medical expenditure for 2 continuous years (*n* = 103) were set as occurrence households and non-occurrence households were selected by PSM for households with three times the average medical expenditure. The occurrence households had US$ 2,275 less average annual income than their comparative group; the income of occurrence households was US$ 2,698 lower than the non-occurrence group in 2009 after the occurrence of continuous high medical expenses. We set 50 households spending twice the average medical expenditure for 2 continuous years as occurrence households and they had on average US$ 3,128 less than their comparative group. After the occurrence of continuous high medical expenditure in 2007–2008, the income of occurrence households was US$ 3,598, US$ 3,198, and US$ 3,244 lower than the non-occurrence group in 2009, 2010, and 2011, respectively.

**Table 5:** Income difference in recurring high medical expenditure

			Unit: USD
Variables	Estimate^§^	Std. Error	*p*-value
≥ at twice the average medical expense (*n* = 412)			
Year (reference: 2008)			
2009	622	52.2	0.2759
2010	1,041	87.7	0.1513
2011	1,123	92.4	0.1174
Case (reference: non-occurrence household)			
Occurrence household	−2,275	111.5	0.0153
Interaction term (year x Case)			
2009	−2,698	124.6	0.0269
2010	−1,143	77.4	0.1695
2011	−911	51.3	0.3295
≥ at three times the average medical expense (*n* = 200)			
Year (reference: 2008)			
2009	2,273	136.4	0.0758
2010	1,775	138.6	0.1715
2011	1,951	117.6	0.0772
Case (reference: non-occurrence household)			
Occurrence household	−3,128	173.5	0.0348
Interaction term (year x Case)			
2009	−3,598	157.2	0.0151
2010	−3,198	159.8	0.0332
2011	−3,244	134.4	0.0104

§ Adjusted odds ratio from multiple regression analysis with all of the variables in Table 1

## Discussion

Health is major factor of human resources and statistically affects economic growth [31]. Therefore, the ability to promote good health and minimize the negative effects of catastrophic medical expenditure is a productive investment in the nation. Since severe disease continues to grow alongside the ever-increasing lifespan of humans, healthcare spending will increase and income disparity and instability of labor market employment will become more serious in the future. Accordingly, this research examined the direct burden of household medical expenses on income in order to estimate problems related to high medical expenditure incurred by households. We stratified the levels of burden according to multiples of average annual medical expenses for high medical expenditure groups. We designed a quasi-experimental study to observe difference between households that otherwise have similar features.

In summary, income differences resulted from spending above three to four times the average medical expenses of typical households for a 2-year period. The data shows a greater reduction of income in occurrence households that spent five times more than the average medical expenses than non-occurrence households during a 3-year period. In addition, the income of households decreased in correlation with medical expenditure of more than two or three times the average medical expense for 2 consecutive years.

A previous study related to our findings examined the effects of catastrophic health expenditure on household impoverishment in South Korea [32]. The results showed that households with high medical expenses economize their expenditure, excluding medical costs, as well as loss of income earned by the unhealthy. In other words, income earned, food expenses, and savings of households decreased and their debt increased after the occurrence of high medical expenses. This response may be explained by counter-strategy to overcome the problems; the households have coped through strong will to survive. Since medical expenses have an involuntary characteristic, household members’ quality of life would be decreased by reducing other expenditure and the uncertainty effects may last for a long time. Thus, the government needs to make progress in strengthening its benefit policy by examining the characteristics of people in detail. The dependent variable of this study is disposable income, which mostly consists of earned income, business income, and property income. There is a possibility that income reduction would be affected by changes in economic status. In other words, a change in employment status or unemployment caused by catastrophic medical expenses of households could worsen economic conditions by having a negative impact on total household income. One of the methods to reduce these risks may be income indemnity insurance, which provides benefits for loss if the insured cannot obtain jobs owing to disease. Income indemnity insurance is provided through private supplementary insurance in many countries (e.g., the United States, Netherlands, France, and Belgium) and it may be possible to implement this system in South Korea [26]. While private supplementary insurance has positive effects on improving access or achieving catastrophic financial protection [33, 34], low-income individuals are less likely to receive such positive effects because they may not be able to afford such insurance. In addition, the insurance could cause moral hazard. If insurance benefits are equal to earned income or higher, the insured might want to continue being unemployed rather than to return to work. The government needs to prevent private insurers from encouraging such negative practices by monitoring them.

This research has some limitations. First, the rate of individuals with private health insurance (PHI) was 65.4 % in 2012, and the rate of households that had at least one member carrying PHI was almost 86.6 % [35]. PHI plans in Korea are typically divided into two kinds: fixed-benefit versus indemnity plans. Fixed-benefit plans pay a lump-sum amount once enrollees are diagnosed with specific diseases, such as cancer. On the other hand, indemnity plans cover actual medical expenses and include both co-payments under the NHI and out-of-pocket payments for services not covered by the NHI (e.g., private room charges), which often become huge burdens for patients. While a recent increase in the rate of households with PHI is expected to relieve the financial burden for those with chronic diseases, this study was unable to incorporate the potential effect of having PHI on medical expense difference because the information was not available. Therefore, this limitation should be acknowledged when interpreting the results of this study. Second, we did not distinguish whether the person or people responsible for high medical expenses in the household were the householder or member of the household. Generally, household income is most affected by the householder (assumed to be the wage earner) because householders threatened by high medical expenditure could bear a bigger burden than household members. We did not consider this factor because of insufficient information.

## Conclusions

This study shows that the occurrence of high medical expenditure may affect household income. This could cause a vicious circle in the household economy because household income reduction could lead to the possibility of reduced health expenditure. Moreover, although support focused on medical expenditure is important, income indemnity insurance policies need to be considered in the long term to protect people at risk of high medical expenditure due to chronic or severe illnesses. Owing to limited resources, households in financial difficulty resulting from high medical costs need to be prioritized in any policy to strengthen benefits. This research indicates that the Korean government should support people who desperately need help with a reasonable and sustainable plan.
